# Heterogeneity and plasticity of tissue-resident memory T cells in skin diseases and homeostasis: a review

**DOI:** 10.3389/fimmu.2024.1378359

**Published:** 2024-05-08

**Authors:** Guomu Liu, Ziyue Wang, Shanshan Li

**Affiliations:** ^1^ Department of Dermatology and Venereology, The First Hospital of Jilin University, Changchun, China; ^2^ Key Laboratory of Organ Regeneration & Transplantation of Ministry of Education, The First Hospital of Jilin University, Changchun, China

**Keywords:** heterogeneity, plasticity, tissue-resident memory T cells, psoriasis, vitiligo, melanoma

## Abstract

Skin tissue-resident memory T (Trm) cells are produced by antigenic stimulation and remain in the skin for a long time without entering the peripheral circulation. In the healthy state Trm cells can play a patrolling and surveillance role, but in the disease state Trm cells differentiate into various phenotypes associated with different diseases, exhibit different localizations, and consequently have local protective or pathogenic roles, such as disease recurrence in vitiligo and maintenance of immune homeostasis in melanoma. The most common surface marker of Trm cells is CD69/CD103. However, the plasticity of tissue-resident memory T cells after colonization remains somewhat uncertain. This ambiguity is largely due to the variation in the functionality and ultimate destination of Trm cells produced from memory cells differentiated from diverse precursors. Notably, the presence of Trm cells is not stationary across numerous non-lymphoid tissues, most notably in the skin. These cells may reenter the blood and distant tissue sites during the recall response, revealing the recycling and migration potential of the Trm cell progeny. This review focuses on the origin and function of skin Trm cells, and provides new insights into the role of skin Trm cells in the treatment of autoimmune skin diseases, infectious skin diseases, and tumors.

## Highlights

- Tissue-resident memory T (Trm) cells are a group that remain in the tissue for long periods of time and are able to stay out of the peripheral circulation for months or even years.
- Skin Trm cells can be expanded from the original Trm source or developed from Trm precursors (Tcm cells, Tem cells, Tmm cells, etc.).
- CD4^+^ Trm or CD8^+^ Trm cells play a harmful role in autoimmune related skin diseases such as psoriasis and vitiligo, while CD8^+^Trm cells play a protective role in tumor diseases such as melanoma.
- Skin Trm cells have the possibility of redifferentiation when they are encountered with pathogens or in a stable state. Mainly CD4^+^ Trm cells can migrate to the remote skin or enter the circulation.
- The redifferentiation and migration of skin Trm cells have considerable prospects in the treatment of various skin diseases.

## Introduction

The main components of skin are generally divided into the epidermis, dermis, and subcutaneous fat area. The basement membrane is the boundary between the epidermis and dermis. The skin is the first barrier to provide robust immune protection against invading pathogens in humans and many other organisms. It relies on an immunomodulatory network of innate and adaptive immune cells and many resident populations (1). The number of T cells infiltrating the skin is much higher than that in peripheral blood; memory T cells account for most of these cells including effector T (TEFF) cells, naive T cells, memory T cells and exhausted T cells (2). In the context of encountering pathogens in barrier tissues, dendritic cells (DCs) initiate the presentation of antigens to naive T cells through lymph node drainage, resulting in the activation, proliferation, and differentiation of naive T cells into effector T cells. Previous research has indicated that following the resolution of inflammation or infection, a small proportion of effector T cells differentiate into memory T cells and probably inhabit the infiltrated tissue, subsequently undergoing local exit or apoptosis (3). However, investigations by Gebhardt et al. utilizing herpes simples virus (HSV)-infected mouse models have identified a specific subset of effector memory T cells in peripheral tissues that persist within the same tissue for an extended period without recirculation after the infection has been eradicated, which subsequently developed into tissue-resident memory T (Trm) cells. They cannot be recycled after local or systemic viral infection and persist after transplantation of infected skin to the recipient (4–6). This results in the emergence of a population of Trm cells. In recent years, Trm cells have been found to reside in lymphoid and non-lymphoid organs for a long time. Traditionally, Trm cells in the skin exhibit prolonged survival for months or even years and reside at the site of the initial antigen encounter. The population of these cells can increase with host age. In the absence of antigenic stimulation, Trm cells can persist in peripheral tissues such as the skin and the female reproductive tract. During this time, they fulfill the role of tissue surveillance, extending dendritic projections within the local tissue to actively search for antigens (7). Upon reactivation by a specific antigen, Trm cells cease their motility and undergo rapid proliferation within the epithelial barrier tissue. Guided by the local tissue microenvironment, Trm cells promptly initiate a secondary immune response and differentiate into effector T cells, providing swift and effective protection against a potential secondary antigenic assault on the tissue (8). Notably, among the diverse array of memory T cells present in the skin, Trm cells occupy a distinct niche with a unique transcriptional profile and phenotype compared to those of other memory cells (9). Multiple studies have identified the expression of CD69 or CD103 as a notable characteristic of these cells, facilitating their tethering to the tissue and inhibiting their egress from the tissue. However, recent studies have suggested that the tissue residence of Trm cells is reversible (10). Trm cells exposed to secondary antigenic stimulation can differentiate into other types of memory cells. For example, the knockdown of CD69 in CD4^+^ Trm cells promotes cell efflux from the skin, and these Trm cells not only reenter the blood circulation but also retain tissue propensity and recolonize secondary skin sites. In a mouse model, CD8^+^ Trm cells were found to proliferate in draining lymph nodes and become circulating memory cells after antigen encounters (11). Thus, Trm cells are found in various non-lymphoid tissues, including the skin, and may reenter blood and distant tissue sites in response to recall, revealing the recycling and migratory potential of Trm cell generation (12).

This review examined a range of memory T cells found in the skin, focusing on Trm cells. Upon entering the skin, multiple subpopulations of memory T cells can differentiate into Trm cells, which exhibit diverse phenotypes in both healthy and diseased states. Furthermore, we will review the delicate balance between Trm cell settlement and recycling, summarize the crucial role of Trm cell plasticity in immune responses, and propose novel hypotheses regarding the involvement of this cell type in various diseases.

## Different types of memory T cells exist in the skin

There are approximately 20 billion T cells in healthy human skin, most of which are CD45RO^+^ memory T cells (up to more than 80%) (13). Memory T cells are usually divided into central memory T (Tcm) cells, Trm cells, effective memory T (Tem) cells and migrating memory T (Tmm) cells. Tcm cells can circulate between the bloodstream and secondary lymphoid organs (SLOs). CD62L and CCR7 are highly expressed in this group of cells, which allows them to maintain their circulatory capacity, similar to the characteristics of naive T cells. Upon encountering pathogen stimulation again, Tcm cells can be mobilized to lymphoid tissues or inflammatory sites beyond lymphoid tissues. These cells undergo rapid proliferation and differentiation into secondary effector T cells, which effectively combat pathogen invasion (7, 14). On the other hand, Tem cells can migrate between the bloodstream and non-lymphoid tissues (NLTs). These cells exhibit a strong inclination toward peripheral tissues and are characterized by low expression of L-selectin and CCR7 (15). Tem cells have primary effector functions and can quickly reach infection sites to form highly protective effector cells after encountering pathogens; thus, they are important for the formation of Trm cells and for the maintenance of Trm cells (9). Moreover, Tem cells are thought to be capable of patrolling the interior of NLTs, but their specific function remains to be elucidated (16). In recent years, the concept of Trm cells, a group of cells that settle in the local tissue and are difficult to migrate, has emerged. Trm cells play a central protective role against NLTs; thus, most memory T cells in NLTs are Trm cells (17). They provide rapid and effective protection against pathogen invasion in barrier tissues. Healthy human skin contains CD4^+^ and CD8^+^ Trm cell populations that are more enriched in the epidermis than in the dermis. These groups of cells play essential roles in integrated memory responses and cutaneous immune responses (18). They present in small numbers in the circulatory system and in SLOs. Tmm cells are the most common CLA^+^ memory T cell population in human blood. These cells exhibit high expression of CCR7 but low expression of L-selectin (19). The characteristics of these cells are between those of Tcm cells and Tem cells, and they have effector functions. However, Tmm cells appear to be excluded from lymph nodes that drain nonskin tissues. They can migrate from the skin to lymphatic vessels and into the skin-draining lymph nodes, a phenomenon not observed in other tissues (20).

The majority of memory T cells in healthy skin are phenotypic CD62L^−^CCR7^−^Tem cells. Tcm cells are present in small amounts in resting skin T cells, and Trm cell proportions can range from 20 to 60%, indicating that the percentage of Trm cells is highly variable in healthy individuals (21). Tissue-specific CD4^+^/CCR7^+^ Tcm cells and CD4^+^/CCR7^–^ Trm cells protected healthy human skin on most occasions, with the latter being significantly more protective. A recent study allergic contact dermatitis showed that TCR complementary-determining region 3 (CDR3) sequences are present in both cutaneous Trm cells and Tcm cells and that the generation of these two cell types in the skin may be related to specific priming signals from dendritic cells (22). A clinical experiment of alemtuzumab in treating leukemic cutaneous T-cell lymphoma (L-CTCL) revealed that Tcm cells and Tmm cells accounted for approximately one-third of the total cutaneous T cells. They circulate between the skin and peripheral blood and are closely linked to the development of the clinical symptoms of the disease. However, skin-resident CCR7^–^/CD69^+^ T cells are unaffected by alemtuzumab treatment, and this population can exert effector functions during disease development (23). Therefore, a variety of memory cells mediate the immune response of the skin, but Trm cells play a significant role in this process.

## The origin of Trm cells in the skin

During the immune process of T-cell response, the fate of each T-cell is determined by the reception and release of different signals. They can undergo apoptosis or differentiation into memory T cells of different phenotypes after an immune response (24). When naive T cells encounter homologous antigens, they activate and proliferate in draining lymph nodes. This results in a corresponding population of effector T cells being able to specifically clear infected cells (25).

A small proportion of effector cells do not undergo apoptosis after pathogen clearance and instead differentiate into memory T cells (9). Skin antigen contact plays a vital role in the developmental trajectory of skin Trm cells. In the secondary immune response, the reactivation of Trm cells can recruit antigen-nonspecific memory T cells to the site of antigen encounter, thereby resulting in effector functions (26, 27). Recent studies have shown that the propensity of various T cells to produce Trm cells is uneven in the effector pool (28). Some studies have used lineage tracking tools to label naive T cells, and labeled TEFF cells, circulating memory T (TCIRCM) cells, and Trm cells have been obtained through mouse skin vaccines *in vivo* (29). The majority of naive T cells migrated into inflamed tissues and underwent differentiation into TCIRCM and Trm cell lineages (84.8%). Researchers have shown a specific subset of circulating TEFF cell clones has a high potential to develop into Trm cells. This suggests that the inclination toward Trm cell formation is acquired prior to tissue entry and that genes associated with Trm cell fate are abundantly expressed. Upon encountering an antigen again, these cells establish themselves as Trm cells (30). Nevertheless, there is also evidence indicating that Trm cells in tissue-draining lymph nodes are biased toward the skin due to their dependence on chemokines, and they do not necessitate antigen encounters. Moreover, CD8^+^ T cells recruited by this route can be present in the skin for more than two months (31). However, little is known about the origin of Trm cells in the skin, that is, the origin of Trm cell precursors. The development of Trm cells is tissue-specific, but the underlying mechanism remains. Multiple subsets of memory T cells have been reported to initiate their differentiation program to become Trm cells, upon encountering homologous antigens in the skin (32, 33).. Among all CD45RO+ memory T-cell subsets, T cells are specifically recognized during maturation by high expression of skin lymphocyte-associated antigen (CLA) and migrate to the skin to form Trm cells (34, 35).. In the case of viral skin infections, CD69^+^KLRG1^−^ effector T cells are highly enriched in the early epidermis after the onset of T-cell infiltration, and the proportion of KLRG1 effector T cells is significantly higher in the spleen and blood than in the skin (36). Further support for circulating Trm cell precursors was provided by a study on Trm cell formation, in which CD127, which is present in humans and is the α chain of IL-7R, was identified. The interaction between IL-7 and CD127 plays an important role in the differentiation and maintenance of T-cell homeostasis (37). It is highly expressed in various autoimmune and inflammatory skin diseases. CD127 is a well-known marker of memory precursor cells. The generation of long lived T-cells is closely related to IL-7 and KLRG1^–^/CD127^+^ T cells are more likely to produce Trm cells in the skin (11). CD8^+^ Tcm cells have been shown to persist as a significant tissue-resident population after pathogen clearance in the skin and to differentiate into functional CD69^+^CD103^−^Trm cells. An elegant study revealed that Tcm cells persist longer in circulation and enter the skin in more significant numbers as Trm cell precursors; these cells are the most dermatophilic and simultaneously produce high numbers of Trm cells and can complement other memory T-cell subsets (20, 38). CCR7^+^ L-selectin^+^ Tcm cells are the most potent precursors of Trm cells in human skin, and this population of cells has high potential to develop into Trm cells. However, the most efficient cell population for Trm cell transformation was Tem cells, which exhibited increased CXCR3 levels and produce interferon-γ (IFN-γ)^+^ Trm cells. In addition, Trm cells produced by migrating memory T cells preferentially express IL-17A (20, 39), and in psoriasis, CD8^+^ Trm cells that produce IL-17A may constitute a pathogenic group in the skin (38).

## Phenotype and localization of cutaneous Trm cells

**Figure 1:**
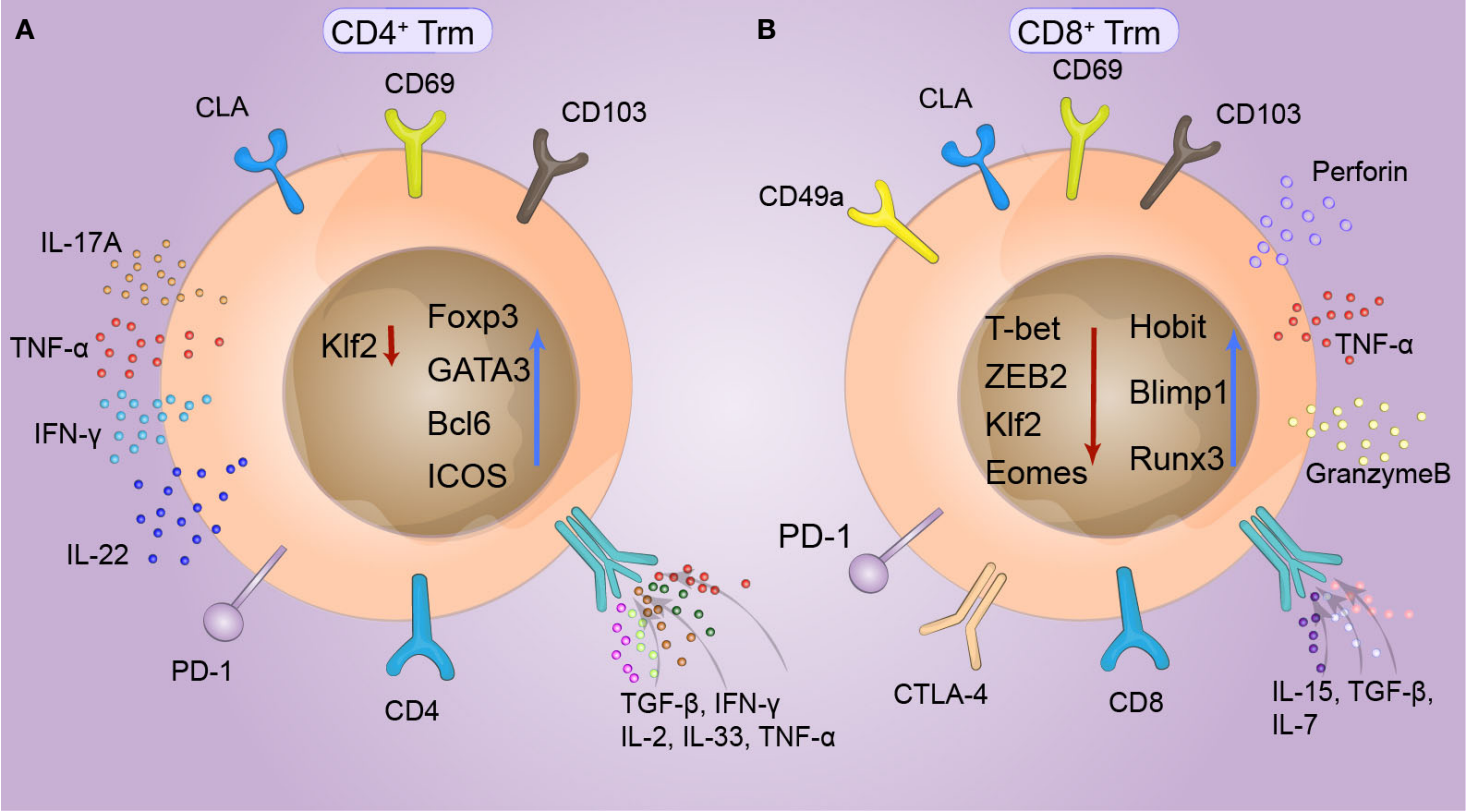
Phenotype of cutaneous tissue-resident memory T (Trm) cells. CD69 and CD103, which are expressed to varying degrees in both CD4^+^ and CD8^+^ Trm cells, are specific markers of skin Trm cells and can specifically limit the retention of T cells. Skin lymphocyte-associated antigen (CLA) is highly expressed in T-cell subsets with a special propensity for binding to skin tissue and is thus recognized by the skin. At the same time, the expression of KLF2 can be temporarily downregulated in response to antigens. **(A)** The survival of CD4^+^Trm cells requires the intrinsic expression of Bcl6 by T cells and continuous signaling through ICOS, which is specifically upregulated in CD4^+^Trm cells. FOXP3 and GATA3 are expressed in T helper cells. IL-17A, IL-22, and IFN-γ are produced by CD4^+^Trm cells in healthy human skin, while TGF-β upregulates CD103 expression in CD4^+^Trm cells, and IL-2, IL-33, IFN-γ, and TNF-α upregulate CD69 expression in CD4^+^ Trm cells. **(B)** The transcription profiles of CD8+Trm cells, including Blimp1, Runx3, and Hobit cells, differed from those of Tcm and Tem cells. Hobit is specifically upregulated together with Blimp1 in CD8^+^Trm cells. The key factors of the transcriptional regulation pathway, T-bet and ZEB2 are synergically downregulated under stimulation by local TGF-β signaling, promoting tissue retention. CD8^+^ Trm cells also exhibit phenotypes similar to those of depleted T cells, including upregulation of a series of immune checkpoints, such as PD-1, LAG-3, TIM-3, and T-cell immune receptors (TIGIT). CD49a is upregulated in CD8^+^ Trm cells, which can produce interferon (IFN-γ), perforin, and granulocase B under appropriate stimulation conditions.

The composition of Trm cells in the entire skin layer differs under stimulation by different antigens in the skin. Trm cells share a variety of common surface tissue markers and transcriptional signatures, which play crucial roles in Trm cell function (40). Two important surface markers of cutaneous Trm cells are CD69 and CD103. CD69 and CD103 were found on T cells but at different levels and with different dependent factors. CD69^+^CD103^−/+^ T cells constitute more than 70% of human skin T cells (41). CD69 is generally considered a marker of TCR-mediated activation, and skin Trm cells constitutively express CD69. CD69 downregulated S1P1 and decreased the expression of ‘egress’ sphingosine-1-phosphate receptor 1 (S1P) (42) to restrict S1PR1-mediated tissue export. Moreover, the expression of the transcription factor KLF2 can be transiently downregulated in response to antigens, thereby reducing the expression of its target gene, S1PR1. These mechanisms limit the efflux of memory cells from tissues, suggesting that S1P1 knockdown can lead to the long-term residency of Trm cells (43). CD103, the alpha chain of the α_E_β7 integrin, is induced by transforming growth factor-β (TGF-β) and binds to E-cadherin on epidermal cells in peripheral tissues, where it specifically restricts T-cell retention. Many researchers consider that the expression of CD103 is not a critical factor for skin Trm cell residency, and memory T cells with CD103^-^ have also been found to have residual abilities. *Yersinia pseudotuberculosis* infection in the oral cavity induces the generation of CD103^+^ or CD103^−^Trm cells in the lamina propria (44). CD49a is the α-subunit of the α1β1 integrin receptor, also known as very late antigen 1 (VLA-1), and binds collagen IV enriched in the basement membrane separating the epidermis and dermis. CD49a expression is limited to only 15% of human skin-derived T cells, which may determine the cytotoxic function of Trm cells. Trm cell differentiation often depends on functional changes in this population of cells. T-cell phenotypes include the production of IL-17A and the upregulation of FOXP3 in CD4^+^ T cells, and the upregulation of CD49a, CXCR3, and CXCR6 and the production of IFN-γ in CD8+ T cells (45). Under appropriate stimulation, CD8^+^Trm cells with high CD49a expression had a higher ability to produce IFN-γ, perforin, and granzyme B (46, 47). The chemokine receptors CCR4, CCR8, CCR10, CXCR3, and CXCR6 are considered critical chemokine receptors for skin Trm cells. CCR8 is expressed in approximately 50% of Trm cells and is rarely expressed in Tcm cells (**Figure 1**).

The transcriptional profiles of Trm cells differ from those of Tcm cells and Tem cells, including the those of transcription factors Blimp1, Runx3 and Hobit (48). Blimp1 and Hobit are jointly and explicitly upregulated in Trm cells, promoting their retention in epithelial barrier tissues, and these two transcription factors have a synergistic effect on Trm cell development (49). In addition, the depletion of S1PR5 enhances the formation of Trm cells. S1PR5 plays a crucial role in T-cell infiltration and migration from peripheral organs and is downregulated in Trm cells. However, T-bet and ZEB2, which are critical factors in the S1PR5 transcriptional regulation pathway, are synergistically downregulated in Trm cells treated with S1PR5 under the stimulation of local TGF-β signaling (50). At the same time, a variety of tumor-associated Trm cells also exhibit phenotypes similar to those of depleted T cells, including upregulation of a series of immune checkpoints, such as TIM-3, LAG-3, PD-1 and T-cell immune receptor (TIGIT) (51). The survival of CD4^+^Trm cells requires T cells to express Bcl6 internally and continuously transmit signals through ICOS and P2X7R. Thus, these two transcription factors are specifically upregulated in CD4^+^Trm cells (**Figure 1**).

### CD4^+^ Trm cells

CD4^+^ T lymphocytes are vital components of adaptive immunity. They can play an important auxiliary role in the function of innate cells, CD8^+^T cells and B cells in the presence of various pathogens (43), especially Candida infection and Leishmania infection. In peripheral CD4 T cells, B-cell interactions lead to upregulation of the transcription factor Bcl6, which also inversely supports the differentiation of memory T cells (52). Similar to those of circulating CD4^+^ memory T cells, the mechanism underlying the formation and diversity of memory CD4^+^T cells are still unknown; therefore, extensive research on tissue-resident lymphocytes has focused on CD8^+^ Trm cells. CD4^+^ Trm cells typically gather in lymphoid tissue, and they interact with antigen-presenting cells upon re-encounter with pathogens. However, recent investigations have revealed a significant disparity between the numbers of CD4^+^ and CD8^+^ T cells within the T-cell population residing in the skin (53). CD4^+^ T cells predominantly populate the dermal and epidermal compartments in humans, whereas in mouse skin, CD4^+^ T cells are primarily concentrated in the dermis (54). In healthy human skin, the proportion of CD4^+^ T cells in the two major layers of skin is approximately 75%, with CD69^+^CD103^+^Trm cells accounting for 50% and 25% of the T cells in the two layers, respectively. Among these, CD4^+^ CD69^+^ CD103^+^ T cells are the most crucial resident population in the dermis. These cells exhibit specificity toward Candida albicans and Staphylococcus aureus and exhibit high expression levels of tumor necrosis factor-α (TNF-α), IL-22 and IFN-γ (55).Due to their limited proliferative capacity, CD4^+^ CD69^+^ CD103^+^ T cells undergo rapid apoptosis when isolated from the skin (56, 57).

Conversely, CD69^+^CD103^–^ Trm cells are present in entire skin layers, constituting 20% and 40% of the T cells in the epidermis and dermis, respectively (54). These cells have a more robust proliferative capacity than double-positive cells; however, their effector function is relatively poor (58). In the dermal compartment, helper T (TH) cells were predominant compared to CD8+T cells, and the expression of CD103 was also relatively low compared to that in the epidermis. Consequently, CD4^+^Trm cells exhibit enhanced migratory capacity but relatively diminished resident ability. In the skin of healthy mice, CD4^+^T cells establish a dynamic equilibrium between migration and circulation with the circulating T-cell pool (59). Nonetheless, heightened skin inflammation or infection leads to augmented recruitment of CD4^+^T cells to the skin and their retention in the dermis. This results in the development of a CD69^+^CD103^+^ CD4^+^ T-cell phenotype embedded within the skin, which possesses potent effector functions (15). The adhesion of CD4^+^T cells to keratinocytes and the upregulation of CD103 expression are facilitated by TGF-β.*In vitro*, TGF-β upregulated the expression of CD103, and TNF-α, IL-33 and IFN-γ upregulated the expression of CD69 (**Figure 1**). These cytokines and surface markers affect cutaneous CD4^+^ Trm cell tissue retention more than antigen recognition (53, 60). CD4^+^ Trm cells can supersede innate immunity upon re-exposure to antigens and exert a direct effector effect. However, the phenotypic and resident characteristics of CD4^+^ Trm cells during infection remain unclear.

### CD8^+^ Trm cells

CD8^+^ Trm cells are located mainly in the epidermal layer of the skin and the basement membrane between the dermis and epidermis. Skin CD8^+^ Trm cells replace the original niche occupied by γδ T cells in the epidermis, enabling their stable existence for several years (61). These cells typically express surface markers such as CD69 and CD103 and have a core transcriptional signature distinct from that of circulating T cells. In the epidermis, CD8^+^ CD69^+^ CD103^+^ Trm cells are a well-characterized subtype; however, at present, CD103 and CD69 are no longer considered the only identified markers of Trm cells. For example, after skin infection with herpes simplex virus 1 (HSV1), CD8^+^ Trm cells in the epidermis are predominantly CD69^+^ CD103^+^ VLA1^+^ CD62l^-^ CD122^-^ and exhibit strong effector function. In contrast, dermal Trm cells are CD103^-^ but have a high proliferative capacity. Therefore, CD8^+^ CD103^+^ Trm cells do not proliferate in large quantities, but CD103^+^ Trm cells are functionally superior to CD103^-^ Trm cells (7). In CD8^+^ Trm cells from mice, the responsiveness to TGF-β signaling is dependent on the suppression of T-box transcription factors, namely T-bet and Eomes, resulting in the upregulation of CD103 expression (62). Thus, the activation of TGF-β is mediated by the integrins avb6 or avb8 on keratinocytes and is regulated by T-box transcription factors. IL-15 plays a crucial role in maintaining the homeostatic proliferation and longevity of CD8^+^ memory T cells. However, only low levels of T-bet expression can sustain the expression of IL-15R (63). Hence, T-bet and Eomes, which belong to the T-box family, regulate the responsiveness to both TGF-β and IL-15 signals, consequently influencing the normal development and function of CD8^+^ Trm cells (64) (**Figure 1**). The requirements for establishing Trm cells were described in a study of skin infections by Jiang et al., who specifically identified ovalbumin (OT-I) and vaccinia virus expressing ovalbumin (VACV) using an adoptive transfer assay (65). The entry of CD8 T cells into the skin is not dependent on the helper function of CD4^+^T cells or the expression of IFN-γ, which is different from what occurs in other epithelial tissues. The expression of CD49a is limited to CD8^+^ T cells and is present only in epidermal CD69^+^CD103^+^ T cells (65).

## Trm cell phenotypes in skin diseases

**Figure 2:**
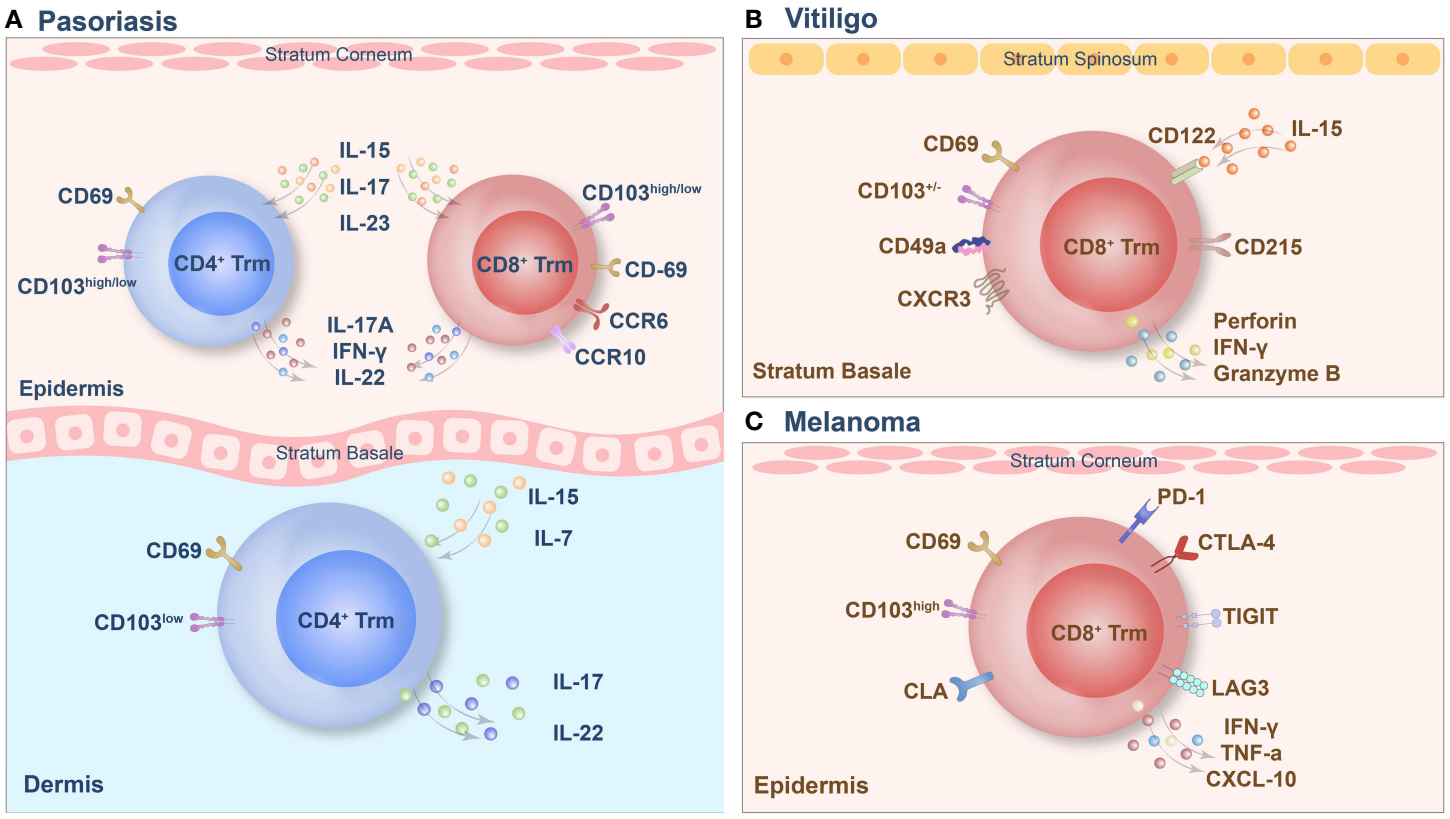
The tissue-resident memory T (Trm) cell phenotypes of psoriasis **(A)**, vitiligo **(B)** and melanoma **(C)**.

Memory T cells in different tissues exhibit tissue specificity in pathological processes, whereas Trm cells in the skin can serve as a “double-edged sword”. On the one hand, they can contribute to disease persistence and recurrence, acting as a “demon”. On the other hand, in certain tumors such as melanoma, Trm cells can also act as “angel”, where their activation in response to tumor proliferation leads to the production of effector functions (66). Thus, Trm cells play dual roles with both beneficial and detrimental effects. Therefore, Trm cells play a vital role in various diseases. The Trm cell phenotypes of 11 types of diseases are briefly summarized in **Table 1**. Here, we discuss vitiligo, psoriasis, and melanoma in the following section (**Figure 2**).

**Table 1:** Trm cell phenotypes in various skin diseases.

Disease	Epidermis	Dermis	Correlation factor	Reference
Psoriasis	CD4^+^CD69^+^CD103^-^ (+++), CD4^+^CD69^+^CD103^+^ (+), CD8 + 103^+^CD49a^-^ (+++), CD8 + 103 + 69^+^ (+)	CD8^+^CD69^+^CD103^-^, CD8^+^CD69^+^CD103^+^	IL-17A, IL-15, Il-22	(67)
Vitiligo	CD8^+^CD49a^+^CD69^+^CD103^-^ (+++) CD8^+^CD49a^+^CD69^+^CD103^+^ (+)		Il-15 、IFN-γ	(1, 68)
Melanoma	CD8^+^CD69^+^CD103^+^CLA^+^		IFN-γ and TNF-α, CXCL10, IL-17	(69, 70)
Cutaneous lupus erythematosus (CLE)	CD8^+^CD103^+^	CD8^+^CD103^+^		(71)
Atopic dermatitis		CD4^+^CD69^+^, CD3^+^CXCR4^+^CD69^+^ (+++), CD8^+^CD69^+^ (+)	IL-4, IL-13, IL-17 and IL-22 IFN-γ	(72)
Pemphigus	CD4^+^CD69^+^CCR7-		IFN-g, IL-4, IL-17A, and IL-21	(73)
Primary cutaneous T-cell lymphomas (CTCL)	CD4^+^CD103^+^ (+++), CD8^+^CD103^+^ (+)	CD4^+^CD69^+^CD103^-^	IL-7 and IL-15	(74)
Pelada		CD8^+^CD69^+^, CD103^+^	IFN-γ, CCL5, CXCL9, CXCL10, STAT1	(59)
Polymorphic light eruption	CD4^+^CD69^+^CD103^+^CD49a- (+++), CD4^+^CD69^-^CD103^+^CD49a^-^ (+)	CD4^+^CD69^+^CD103^+^CD49a^-^ (+++), CD4^+^CD69-CD103^+^CD49a^-^ (+)	IFN-γ and GzmB, IL-15	(75)
Fixed drug eruption	CD8^+^CD45RA^+^CD69^+^		IFN-γ	(76)
Allergic contact dermatitis	CD8^+^CD69^+^ CD103^+^		PD-1, TIM-3, IFN-γ, GzmB	(77)

### Psoriasis

Psoriasis is an autoimmune inflammatory skin disease, the pathogenesis of which involves the disruption of the immune balance of T cells, followed by excessive keratinocyte proliferation (78). Trm cells are highly enriched in active psoriasis lesions and are also found in stable psoriasis lesions (79). CD3^+^ T cells infiltrated extensively throughout the entire skin layers of the lesion and included the CD4^+^ and CD8^+^ subgroups. CD8^+^ Trm cells in psoriasis have recently been found to express CD69, CD103 and CLA and to play a key pathogenic role (54). In psoriatic skin lesions, CD103 is expressed in most epidermal cells by CD8^+^ T cells, which are usually coexpressed with CD69, while this group of cells is negative for CD103 expression in the dermis, and a small number of CD8^+^CD103^+^Trm cells are observed under the dermal papilla (80). A small amount of CD4^+^ Trm cells can infiltrate the epidermis and dermis. Trm cells in the dermis express high levels of CD103, while Trm cells in the epidermis express low levels of CD103 (81). The specific pathogenicity of this group of Trm cells depends on the cytokines they produce, and the key cytokines involved in this process are IL-17 and IL-22. In addition, recruitment of Trm cells is dependent mainly on IL-15 and IL-7 mediated immune responses. CD8^+^CD69^+^CD103^+^Trm cells in the epidermis can express CCR6 and IL-23R, and under stimulation *in vitro*, these cells produce IL-17A and promote the recurrence of skin lesions in the same area via the secretion of IL-17 (38). A subset of CD8^+^ CD103^+^ T cells and CD4^+^CD103^+/–^ T cells also express IL-17A and produce IFN-γ or IL-22. Additionally, CD4^+^CD103^+^ Trm cells are capable of colonizing the epidermis. Interestingly, the population of CD8^+^CD103^+^IL-17A^+^ Trm cells tends to be higher in patients treated with biological agents or systemic therapy (67). In addition, the proportion of CD4^+^/CD8^+^ CD103^+^ IL-17A^+^ Trm cells in the advanced treatment group was significantly higher than that in the nonadvanced treatment group (82). Under inflammatory and homeostatic skin conditions, a population of CD8^+^CCR10^+^ T cells is present but not enriched in psoriatic lesions. These cells express GATA3, FOXP3, and many transcriptional features of Trm cells, including CD103 (79). In addition to T cells, the long-term role of Th22 and epidermal dendritic cells in psoriasis confirms that Trm cells persist in the epidermis of cured skin lesions and can function for several years (83). Therefore, targeting memory T cells in the psoriatic epidermis, particularly CD8^+^ Trm cells, may be a promising approach for treating psoriasis. However, the presence of CD4^+^ Trm cells should not be ignored because they may be an essential factor leading to disease recurrence and persistence.

### Vitiligo

Vitiligo is a skin disease characterized by a high recurrence rate, and existing evidence supports the concept of local immune “memory.” In addition, the recurrence of vitiligo suggests that autoimmune memory may develop within the lesion and contribute to disease relapse (84). The main factor in this immune memory is the formation of Trm cells, which play a vital role in the recurrence of vitiligo. Studies have shown that CD8^+^ Trm cells are predominantly present in the epidermis and dermis of skin lesions in vitiligo patients. However, their numbers are significantly higher in the epidermal compartment than in the dermis (85). In mouse models, approximately 60–90% of melanocyte-specific CD8^+^ T cells in vitiligo patients express the Trm cell markers CD69 and CD103, which are highly enriched in skin lesions. Trm cells can be present for more than a year, and their enrichment sites are mainly at the epidermal/dermal junction, which contains hair follicles with a marked reduction in melanocytes (86). CD8^+^ Trm cell subpopulations expressing CD69, CD103, and CXCR3 were enriched in the epidermal compartments of patients with vitiligo, and they were significantly enriched in stable and active lesions. In lesions, two subsets of tissue-resident memory T (suTrm) cells are present: CD69^+^CD103^-^ Trm cells and CD69^+^CD103^+^ Trm cells. Moreover, CD69^+^CD103^+^ Trm cells are more abundant in the skin of patients with stable vitiligo than in that of patients with active disease (87). CD49a plays a crucial role in vitiligo lesions, and this group of cells exhibits strong cytotoxicity due to the constitutive expression of perforin and granzyme B in CD8^+^CD49a^+^ Trm cells. IL-15 is also integral in the development of vitiligo, as the CD122 subunit of the IL-15 receptor is expressed on Trm cells in both humans and mice (87). By targeting the IL-15 receptor, long-term blockade of CD122 depletes PMEL Trm cells, while short-term blockade of CD122 also achieves repigmentation by reducing its effector function. CD215, a subunit on the surface of Trm cells that is required for cell activation and cytokine expression, is highly expressed on keratinocytes (87). Recent studies have reported that the maintenance of decolorization of skin lesions in patients with vitiligo is simultaneously associated with autoreactive recirculating memory T (TRCM) cells in the blood, which bind to CXCR3 on the surface of TRCM cells via CXCL9 and CXCL10. Thus, TRCM cells are recruited to the lesion site. Under the synergistic effect of Trm cells and TRCM cells, decolorization is maintained for a long time by the production of IFN-γ and other cytokines. Therefore, simultaneously targeting Trm cells and TRCM cells to block the CXCL9/10-CXCR3 pathway in vitiligo patients may be an effective strategy for treatment (8).

### Melanoma

Trm cells are a critical TIL subset that regulates the immune network in the melanoma microenvironment (88). Mice lacking Trm cell formation are more likely to develop tumors. Trm cells actively inhibit cancer progression, particularly CD8^+^ Trm cells in the epidermal layer. These compounds promote durable melanoma immune homeostasis. In particular, melanoma-specific Trm cells can exert profound inhibitory effects on tumor development independent of circulation before melanoma development (70). Melanoma-specific Trm cells reside in hair follicles primarily depleted of melanocytes. They do not require recirculating central memory T cells or lymph compartment recruitment for maintenance and persist in hair follicles for a long time. This population of cells expresses CD69, CD103, and CLA and can produce IFN-γ and TNF-α. Among these critical factors, the expression of CD103 is required for the formation of CD8^+^ Trm cells in the skin. CD103^+^ CD8^+^ Trm cells play a solid protective role, and the long-term response of melanoma CD8^+^T cells to immunotherapy for more than one year can effectively prevent the reattack of melanoma; thus, CD103-dependent Trm cells play a crucial role in antitumor immunity (89). Another TCR sequence analysis revealed the coexistence of Trm cells in the skin and Tem cells in the blood. This study also revealed the presence of identical clonotypes in both the skin and blood, as found in tumors (69). Furthermore, long-term survival of these T cells for up to nine years, along with high expression levels of IFN-γ and TNF-α in the tumor, skin, and peripheral blood clonotypes, has been demonstrated (69). High levels of these cytokines are sufficient to guarantee a good prognosis in patients with melanoma. It has also been demonstrated that the tumor-associated clonal repertoire is retained mainly in the skin. Trm cells have many similarities to tumors and viral skin infections. They have a protective effect on the skin and can be maintained for an extended period. However, it is worth mentioning that the expression of the immune checkpoints PDCD1, LAGLA3, and TIGIT is higher in tumor-specific Trm cells than in Tcm cells or Tem cells (37). CD8^+^CD69^+^CD103^+^ Trm cells also showed higher PD-1 and lag3 immune checkpoint expression than CD8^+^CD69^-^CD103^-^ cells. At the same time, they also express the first immune checkpoint molecule, CTLA-4, which inhibits the anticancer response of CD8^+^ cells. Between T cells and dendritic cells, CTLA-4 interaction occurs mainly during antigen presentation in the draining lymph nodes. However, CTLA-4 inhibits T cell activation and proliferation by competing with CD28 to block the costimulatory signals required for T-cell activation. The constitutive expression of CTLA-4 suppresses T-cell-mediated immune responses in response to chronic TCR stimulation by infection or malignancy (90). However, the role of this critical checkpoint in Trm cell function remains unclear (37).

## Plasticity of the skin Trm cell

**Figure 3:**
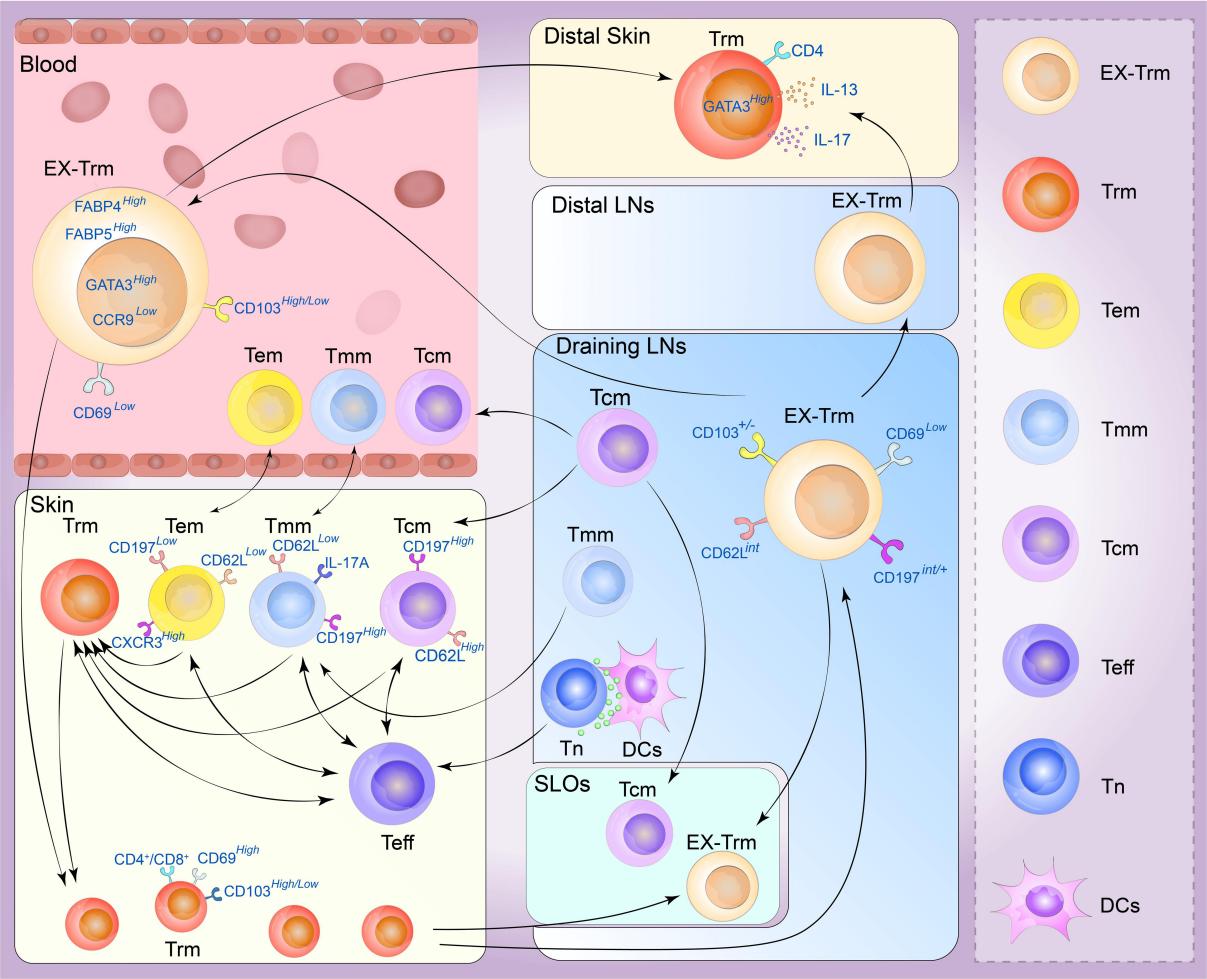
Plasticity of the skin tissue-resident memory T (Trm) cell. Memory T cells are generally divided into four types. Central memory T (Tcm) cells circulate between the blood and secondary lymphoid organs (SLOs) mostly through draining lymph nodes, and can be recruited into lymphoid tissue or to sites of inflammation beyond it. Effective memory T (Tem) cells can migrate between blood and non-lymphoid tissues (NLTs), showing a strong peripheral tissue bias. Tmm cells preferentially patrol peripheral tissues and migrate to lymph nodes and blood. They can migrate to draining lymph from the skin but are excluded from lymph nodes draining non-skin tissue. Trm cells are mainly derived from the differentiation of effector T-cell populations, and the generation of secondary Trm cell populations depends on the proliferation of preexisting Trm cells and a variety of Trm progenitor cells in the skin can also transform into Trm cells under certain conditions. Under stable and inflammatory conditions, skin Trm cells can cross the tissue outlet to reenter the circulation and SLOs, and these cells can form and redifferentiate to other types of memory cells. These Trm cells that flow out of tissues are commonly referred to as ex-Trm cells, which retain dermotropism and its associated transcriptional signatures. Migrating CD4^+^ T cells may exhibit a transitional phenotype during migration and re-enter circulation, distal lymph nodes, and nonspecific skin inflammatory sites through draining lymph nodes. After this group of cells migrated to secondary human skin sites, Trm cells phenotypes could be represented. (Tn, navie T cell; Teff, effector T cell; DCs, dendritic cells; LNs, lymph nodes).

By draining the lymph nodes, naive T cells initiate a primary immune response in SLOs, thereby draining the infected barrier tissue (**Figure 3**). Effector T cells proliferate to acquire effector capabilities before infiltrating local tissues to exert their functions. In the context of the secondary immune response, this process can be conceptualized as an “inside-out” model, where Tcm cells are promptly activated in lymph nodes and subsequently migrate to non-lymphoid tissues (91). In the skin, Trm cells possess the potential to govern local recall responses and exhibit effector functions (92). Interestingly, certain studies have indicated that the reactivation of Trm cells can trigger the recruitment of Tcm cells; however, the *in situ* differentiation of Trm cells can occur independently of antigen stimulation. The generation of secondary Trm cell populations relies on the proliferation of pre-existing Trm cells rather than their derivation from the circulating T-cell pool (93). Hence, Trm cells represent a relatively autonomous population capable of proliferating independently of the main memory or lymphoid tissue. They can even expand local immune surveillance autonomously without the involvement of antigen-presenting cells (10). However, the expansion ability of Trm cells is far lower than that of naive T cells or Tcm cells because of the inherent differences in the proliferative potential of T cells. For this reason, Trm cells are difficult to study *in vitro*, as these cells have difficulty surviving after being separated from the tissue microenvironment (94).

Recent studies have shown that cutaneous Trm cells have novel circulating characteristics in mice and humans, and Trm cells in the NLT, including the skin, exhibit significant levels of developmental plasticity. They can traverse tissue outlets to re-enter circulation and SLOs under both inflammatory and steady-state conditions, and their phenotypes can redifferentiated into various types of memory cells. Gebhardt identified two distinct subpopulations of antigen-specific memory T cells in the skin of patients with herpes simplex virus infection: a subset of CD8^+^T cells that reside in the epidermis and a population of CD4^+^T cells that can rush through the dermis, demonstrating that CD4^+^T cells are more capable of migrating than CD8^+^ T cells (6). Secondary antigen-stimulated Trm cells, particularly those with a CD4^+^ phenotype, have been shown to migrate retrogradely, generate Tcm cells and Tem cells, and retain the biased homing and differentiation potential of Trm cells. In contrast, most skin CD4^+^ T cells are in equilibrium with circulating T cells rather than stably residing in the tissue. In CD45.1 and CD45.2 congenic mice symbiosis experiments, almost half of the skin infiltrating T cells were CD4^+^ Trm cells which express CD69 and CD103, and the cell subtypes exhibited by the co-organisms were very similar, demonstrating that these cells can flow through circulation (90). These tissue-derived Trm cells, called ex-Trm cells, retain dermatophagic and associated transcriptional characteristics. For example, the cutaneous extracellular matrix still expresses high levels of GATA3, cytoplasmic FABP4, and FABP5 but lacks the characteristics of other tissue-derived Trm cells, such as CCR9. According to a previous study (30), these cells can migrate back to the skin area under favorable conditions and opportunities. Klicznik et al. reported that CD4^+^CD69^+^CD103^+^ Trm cells can exit human skin by downregulating CD69 expression. The authors also identified cell populations in the blood and lymph nodes with transcriptional profiles and clonotypes comparable to those of the human skin CD4^+^CD69^+^CD103^+^ Trm cell population, confirming that part of the human skin CD4^+^CD103^+^ Trm cell population can reenter circulation (95). These cells may express an intermediate phenotype (CCR7^int/+^CD62L^int^CD69^–^CD103^+/–^E-selectin ligands^+^) along the way and reenter distal lymph nodes, sites of nonspecific skin inflammation and even the circulatory system through draining lymph nodes (96). After this population of cells migrates to secondary human skin sites, the Trm cell phenotype reappears. This provides new insights into the segmentation of human CD4^+^ memory T cells (96). A recent allogeneic hematopoietic stem cell transplant (HSCT) study revealed host CD4^+^ T cells with a skin-resident phenotype in patients who underwent immune cell reconstitution after HSCT. These cells showed Th2/Th17 characteristics with high GATA3, IL-13, and IL-17 expression. This population of cells is skin-derived and highly similar to cutaneous Trm cells (97). In a unique type of allogenic HSCT environment, host Trm cells form a symbiotic association with donor T cells, resulting in a chimera (98). These chimeric Trm cells can persist in human skin for decades without replenishing the circulation pool. In contrast, a subset of T-cell clones in the patient’s skin and blood showed cross-sharing between tissues and time points. This suggests that tissue injury may stimulate Trm cell activation and retrograde migration and that Trm cell reseeding and inflammatory cell migration can occur in distant organs (88).

According to a lineage-tracing mouse model, CD8^+^ Trm cells also have the potential to form circulatory effector cells and memory cells in the secondary immune response. Hobit^+^ Trm cells demonstrate downregulation of Hobit expression upon encountering antigens, and Hobit^+^ CD8^+^ Trm cells can proliferate in draining lymph nodes. Consequently, they give rise to circulating memory cells following pathogen rechallenge. Moreover, pre-hobit^+^ T cells undergo redifferentiation into CD8^+^ Tem cells upon pathogen rechallenge, and these cells primarily acquire the phenotype of Tem cells (99). However, recent investigations have examined the gene profiles of CD69^+^ CD8^+^ T cells from various human tissues and revealed low to negligible levels of Hobit expression. As a result, further investigations are required to determine whether the differentiation mechanism of CD8^+^ Trm cells in humans corresponds precisely to the findings observed in mice (64). However, another high-throughput sequencing analysis revealed that the methylation status of CpG regions in CD8^+^ Trm cells indicated that CD8^+^ Trm cells could redifferentiate in tissue regions and acquire the corresponding effector functions (100). These results suggest that CD8^+^ Trm cells maintained at the site of local inflammation may also re-engage in systemic memory immune responses, supporting the inside-out characteristic of protective immunity (101). Memory responses can be initiated *in situ* at sites of infection and within the tissue microenvironment in which skin Trm cells are located, and Trm cells have some proliferative capacity to redistribute and even support circulating memory T-cell pools (7).

Some T-cell populations, such as CD4^+^ Trm cells or CD8^+^ Trm cells, retain recycling programs in non-lymphoid tissues. This observation emphasizes mechanisms that enable T-cell migration to non-inflammatory tissues, and the extent of tissue specificity (and associated molecular mechanisms) of these recycling pathways remains a crucial area of research. However, the plasticity of CD4^+^ Trm cells is more vital than that of CD8^+^ Trm cells, and the recycling of CD4^+^ Trm cells has been relatively well reported (102). Since S1P has been shown to promote the efflux of reactivated T cells from non-lymphoid tissues, we hypothesized that these recycled cells retain some of the functional and transcriptional properties of Trm cell precursors, such as Tcm cells or Tem cells (**Figure 3**). The direction of recycling may also be heterogeneous, and the T-bet axis may positively regulate this process. For example, the expression levels of CD62L and CD197 may be higher than those in Trm cells, and Trm cells may also express higher levels of CXCR3 or KLRG1. Further studies are required to determine the long-term migratory behavior of Trm cells, including their potential to reenter the circulation and migrate to distant tissue sites. These investigations may offer insights into the mechanisms of protective immunity in localized areas of the skin (103).

## Discussion

Trm cells can develop from various memory cells, reside in lymphoid and non-lymphoid organs and are not traditionally recycled through the blood. The Trm cells in the skin are heterogeneous, and different cell subpopulations with different surface markers and functional expression levels of correlation factors may be involved in multiple diseases and anatomical conditions (104). CD8^+^ Trm cells are essential for the development of diseases such as vitiligo, psoriasis, and melanoma. CD8^+^ Trm cells can lead to recurrence or difficulty in curing disease through the local settlement of Trm cells. Therefore, Trm cell targeting is an attractive therapeutic strategy. Currently, the definition of Trm cells is constantly changing. Recent studies have shown that Trm cells reside in tissues for a long time. Various Trm cells, including CD4^+^ and CD8^+^, cells can exhibit a high degree of plasticity under steady-state and inflammatory conditions and thus migrate to draining LNs, circulation, distal LNs, and nonspecific skin inflammation sites. In both human and mouse models, CD4^+^ Trm cells are traditionally considered a population with limited residency that is capable of exiting the skin, reentering the bloodstream, and potentially migrating to distant tissue sites (95). The characteristics of CD4^+^ Trm cells display significant variability across tissues, and distinct cues contribute to their establishment and retention. Recent studies have also indicated that CD8^+^ Trm cells can regain Tcm cell and Tem cell phenotypes in mouse models. These findings suggest that the current understanding of Trm cells may not be confined to their settlement solely within local tissues. Whether the residence of Trm cells in various diseases is time-limited remains uncertain, and further investigations are needed to explore whether Trm cells represent an intermediate stage rather than the final destination in the circulation of memory T cells across tissues, lymph nodes, and blood.

Some researchers believe this raises the possibility that the pathology can be distributed to distant tissue sites in the case of harmful effects mediated by Trm cells. For example, in autoimmune diseases involving Trm cells, the potential migration of Trm cells to distant sites could result in dissemination and metastasis to the lesion site, transforming a localized disease into a systemic disease. Alternatively, it progresses from a stable phase to an active phase. However, if the formation of Trm cells is reversible, then more possibilities for Trm cell-related treatment can be obtained. Therefore, to control the spread and recurrence of this disease, targeted Trm cell therapy will achieve improved efficacy. For example, treatment with Mart-1-specific Trm cells is very popular in the treatment of vitiligo. By inhibiting the generation of skin Trm cells, such as anti-IL-15 antibodies, at the lesion site, the recurrence of the disease can be reduced (84). Due to the positive effect of Trm cells on many tumors and infectious diseases, Trm cells can play a good role in disease resistance. The induction of redifferentiation and migration may become a new strategy for treating a variety of tumors using Trm cells and may also increase the targeting of various diseases. For example, Trm cells are a key group of CD8^+^ T cells involved in immune checkpoint inhibitor therapy. The antitumor effect of anti-PD-1 depends on the localization of CD8^+^ T cells at the tumor margin, proving that memory T cells are critical for mediating the anti-PD-1 response. Tumor-specific epidermal CD69^+^ CD103^+^ Trm cells have a sustained protective effect on melanoma patients, especially premelanoma melanoma-specific Trm cells, which can function independently of circulation and have a far-reaching inhibitory effect on tumor development (105). Melanoma-specific Trm cells can persist in the skin for a long time, and the CD8^+^ T cells of melanoma patients exhibit a long-term response to immunotherapy for more than one year. In particular, CD103^+^ CD8^+^ Trm cells can play a key role in antitumor immunity and effectively prevent the reattack of melanoma cells (89). In other diseases, CD8^+^ Tcm cells can migrate into the skin, where they are the first line of defense against subsequent infection after skin vaccinia virus infection has subsided (106). In addition, a colony of IFN-γ-producing Leishmania-specific memory CD4^+^ Trm cells that formed in response to parasitic infection were able to remain in the skin when transplanted into juvenile mice. Their function is dependent on CXCR3, which recruits circulating T cells to the skin to provide optimal protective immunity against Leishmania (107). Therefore, an anti-Leishmania vaccine targeting the generation and amplification of Trm cells will be a hot new strategy in the future.

## Conclusion and outlook

Overall, the resident and recycling functions of Trm cells should be viewed from a dialectical perspective. Skin Trm cells are two-sided. Although Trm cells are associated with the progression of many diseases, they still play a powerful role in the protection against many diseases in the local area. In recent years, the mechanisms of TRM cells in different skin diseases have begun to be revealed. It is clear that TRM cells are strongly involved in the development and recurrence of skin diseases. Further study of its heterogeneity and plasticity will not only enhance our comprehension of diseases, but more importantly, this will facilitate the development of more effective therapeutic approaches.

## Author contributions

GL: Writing – original draft, Investigation, Formal analysis, Data curation. ZW: Writing – review & editing, Visualization, Data curation, Conceptualization. SL: Writing – review & editing, Visualization, Supervision, Funding acquisition.
