# Corrigendum: Heterogeneity and plasticity of tissue-resident memory T cells in skin diseases and homeostasis: a review

**DOI:** 10.3389/fimmu.2024.1460250

**Published:** 2024-07-18

**Authors:** Guomu Liu, Ziyue Wang, Shanshan Li

**Affiliations:** ^1^ Department of Dermatology and Venereology, The First Hospital of Jilin University, Changchun, China; ^2^ Key Laboratory of Organ Regeneration & Transplantation of Ministry of Education, The First Hospital of Jilin University, Changchun, China

**Keywords:** heterogeneity, plasticity, tissue-resident memory T cells, psoriasis, vitiligo, melanoma

In the published article, there was an error in the **Funding** statement. An incorrect funding body and grant number were listed, and a grant number for the National Natural Science Foundation of China was erroneously excluded. The correct **Funding** statement appears below.

**FUNDING**

The author(s) declare financial support was received for the research, authorship, and/or publication of this article. This work was supported by Jilin Provincial Health Special Project (No. 2018SCZWSZX-017) and the National Natural Science Foundation of China (NSFC) (No. 82373492 and No. 81773317).

The authors apologize for this error and state that this does not change the scientific conclusions of the article in any way. The original article has been updated.

